# Caution Concerning Chloroform and Strychnine

**Published:** 1884-12

**Authors:** 


**A Caution Concerning Chloroform and Strychnine.**—Dr. E. Garraway writes to the *British Medical Journal* that a prescription calling for a fluid drachm of solution of sulphate of strychnine, three fluid drachms of spirit of chloroform, and water enough to make a fluid ounce and a half, was put up for a lady, with directions that a teaspoonful was to be taken three times a day in water. On taking the final dose, which tasted unusually hot, the patient was seized with spasmodic contraction of the extensors of the feet and legs, which soon passed off, although alarming at the time. On his being shown the bottle, a drop or two of water was seen remaining in it, and beneath this a globule of chloroform which proved to be highly charged with strychnine. His explation is that more chloroform was ordered than the water would take up, and the surplus, being a ready solvent of sulphate of strychnine, took up a large amount of the salt and fell to the bottom.

